# Scintigraphic features of Morquio's syndrome: a case report

**DOI:** 10.1186/1752-1947-5-42

**Published:** 2011-01-28

**Authors:** Bjoern Kitzing, Kevin C Allman

**Affiliations:** 1 Department of PET and Nuclear Medicine, Royal Prince Alfred Hospital, Missenden Road, Sydney, New South Wales, Australia

## Abstract

**Introduction:**

To the best of our knowledge, we present for the first time the bone scintigraphy findings of a patient with Morquio's syndrome.

**Case presentation:**

A 46-year-old Caucasian man with Morquio's syndrome presented with lower back pain six weeks after a left total hip replacement. A whole body bone scan demonstrated an anthropomorphic skeletal pattern consistent with a mucopolysaccharide storage disease, thereby showing the cause of the patient's pain.

**Conclusions:**

To the best of our knowledge, the bone scintigraphy findings of a case of Morquio's syndrome have never before been published. We present our case report to add to the knowledge we have of this rare disease.

## Introduction

Morquio's syndrome is an autosomal recessive mucopolysaccharide storage disease which is characterized by the inability to metabolize keratin sulphate. It was first described in 1929 by Luis Morquio in Montevideo, Uruguay [1]. He observed the disorder in four siblings in a family of Swedish extraction and recognized the occurrence of corneal clouding, aortic valve disease and the urinary excretion of keratan sulfate. The symptoms associated with Morquio's syndrome are usually noticed between one to three years of age and can include abnormal heart development, abnormal skeletal development, hyper-mobile joints, large fingers, knock-knees, widely spaced teeth, a bell-shaped chest, compression of the spinal cord, an enlarged heart and dwarfism [2-4]. The syndrome is estimated to occur in one in every 200,000 births, with a family history of the syndrome raising one's risk of developing the condition.

## Case presentation

**Figure 1:**
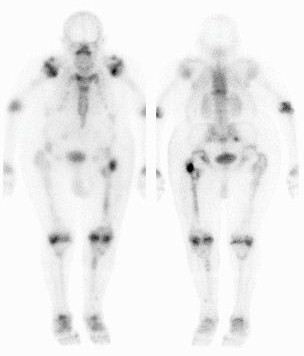
**Anterior and posterior whole body bone scan views of a 46-year-old Caucasian man with Morquio's syndrome complaining of lower back pain**. The anthropomorphic skeletal uptake pattern is consistent with a mucopolysaccharide storage disease.

**Figure 2:**
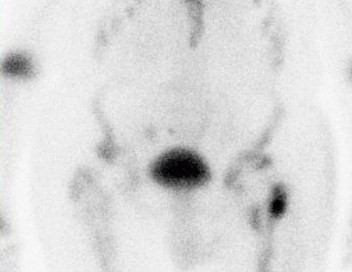
**Localized anterior bone scan view of the pelvis of a 46-year-old Caucasian man with Morquio's syndrome demonstrating bilateral hip replacements**. There is increased peri-prosthetic uptake in the left hip in keeping with recent hip replacement.

**Figure 3:**
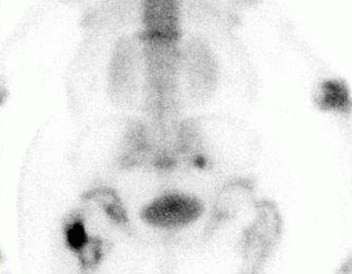
**Localized posterior bone scan view of the pelvis and lumbar spine of a 46-year-old Caucasian man with Morquio's syndrome, three hours after a Tc-99m methylene diphosphonate injection**. Focal increased uptake is seen in the right sacroiliac joint and sacral body, consistent with fractures, and most likely representing the cause of the patient's pain.

A 46-year-old Caucasian man with Morquio's syndrome presented to our nuclear medicine department for a whole body bone scan. He had been complaining of lower back pain for six weeks following a left total hip replacement. He had been referred to our facility for bone imaging by his general practitioner. Whole body anterior and posterior static views were obtained three hours after an injection of 800MBq Tc-99m methylene diphosphonate, demonstrating an anthropomorphic skeletal uptake pattern consistent with Morquio's syndrome. The known radiological features of Morquio's syndrome include short-trunk dwarfism, a shortened neck with the head appearing sunken into the chest, broad flat ilia, widespread degenerative disease and skeletal dysplasia [2]. These features were confirmed on the bone scintigraphy (Figures 1, 2, 3). The scan also showed that he had had both hips replaced. The unusual tracer uptake in the proximal part of both tibias was due to previous shorteningand straightening procedures. There was increased peri-prosthetic uptake in his left hip in keeping with a recent hip replacement (Figure 2). Focal increased uptake was observed in his right sacroiliac joint and sacral body, consistent with fractures, and this was the most likely cause of his pain (Figure 3). There was also increased linear uptake in his lower thoracic spine consistent with an old crush fracture. The bone scan report was sent to the general practitioner with whom our patient had a follow-up appointment.

## Conclusions

To the best of our knowledge, the bone scintigraphy features of Morquio's syndrome have never before been published. The clinical and radiological findings, however, have previously been described [2-4] and we hope that our case report will add to the published literature on this rare condition.

## Abbreviations

MBq: megabecquerel; Tc-99m: metastable technetium-99.

## Consent

Written informed consent was obtained from the patient for publication of this case report and any accompanying images. A copy of the written consent is available for review by the Editor-in-Chief of this journal.

## Competing interests

The authors declare that they have no competing interests.

## Authors' contributions

BK made substantial contributions to the conception and design of the study and drafted the manuscript. BK and KCA both read and approved the final manuscript.
